# Carboxypeptidase E conditional knockout mice exhibit learning and memory deficits and neurodegeneration

**DOI:** 10.1038/s41398-023-02429-y

**Published:** 2023-04-26

**Authors:** Fang-Cheng Fan, Yang Du, Wen-Hui Zheng, Y. Peng Loh, Yong Cheng

**Affiliations:** 1 Key Laboratory of Ethnomedicine of Ministry of Education, Center on Translational Neuroscience, School of Pharmacy, Minzu University of China, Beijing, China; 2 Section on Cellular Neurobiology, Eunice Kennedy Shriver National Institute of Child Health and Human Development, National Institutes of Health, Bethesda, MD 20892 USA; 3 College of Life and Environmental Sciences, Minzu University of China, Beijing, China; 4 Institute of National Security, Minzu University of China, Beijing, China

**Keywords:** Hippocampus, Physiology

## Abstract

Carboxypeptidase E (CPE) is a multifunctional protein with many nonenzymatic functions in various systems. Previous studies using *CPE* knock-out mice have shown that CPE has neuroprotective effects against stress and is involved in learning and memory. However, the functions of CPE in neurons are still largely unknown. Here we used a *Camk2a*-Cre system to conditionally knockout CPE in neurons. The wild-type, *CPE*^flox/−^, and *CPE*^flox/flox^ mice were weaned, ear-tagged, and tail clipped for genotyping at 3 weeks old, and they underwent open field, object recognition, Y-maze, and fear conditioning tests at 8 weeks old. The *CPE*^flox/flox^ mice had normal body weight and glucose metabolism. The behavioral tests showed that *CPE*^flox/flox^ mice had impaired learning and memory compared with wild-type and *CPE*^flox/-^ mice. Surprisingly, the subiculum (Sub) region of *CPE*^flox/flox^ mice was completely degenerated, unlike the CPE full knockout mice, which exhibit CA3 region neurodegeneration. In addition, doublecortin immunostaining suggested that neurogenesis in the dentate gyrus of the hippocampus was significantly reduced in *CPE*^flox/flox^ mice. Interestingly, TrkB phosphorylation in the hippocampus was downregulated in *CPE*^flox/flox^ mice, but brain-derived neurotrophic factor levels were not. In both the hippocampus and dorsal medial prefrontal cortex, we observed reduced MAP2 and GFAP expression in *CPE*^flox/flox^ mice. Taken together, the results of this study demonstrate that specific neuronal *CPE* knockout leads to central nervous system dysfunction in mice, including learning and memory deficits, hippocampal Sub degeneration and impaired neurogenesis.

## Introduction

Carboxypeptidase E (CPE), also known as neurotrophic factor-α1, is a multifunctional protein with many essential nonenzymatic functions in the endocrine and nervous systems [1]. CPE is enriched in mature secretory vesicles and plays a critical role in the biosynthesis of peptide hormones and neuropeptides [2]. CPE also acts as the sorting receptor of many proproteins, including proinsulin, proenkephalin, pro-opiomelanocortin and pro-brain-derived neurotrophic factor (pro-BDNF) [3].

A naturally occurring obesity phenotype mouse mutation, named “fat”, has been mapped to the *CPE* gene [4]. A subsequent study indicated that *CPE*^fat/fat^ and *CPE*-knockout (KO) mice showed similar phenotypes, including infertility and adult obesity [5]. It has been reported that the human *CPE* gene has mutations, including null mutations, mutations that yield truncated proteins and those with key catalytic residues eliminated [6]. Homozygous individuals with severe mutations are morbidly obese and have hypogonadism. In addition, *CPE* missense polymorphism has been found in type 2 diabetes patients. The mutation alters CPE enzyme activity, and patients show early onset of type 2 diabetes [7]. Furthermore, CPE plays an important role in vesicular transport, as shown in hippocampal neurons and synapses [8].

Previous studies have shown that high levels of CPE are expressed in the hippocampus and have a neurotrophic role independent of its enzymatic activity in protecting against stress-induced pyramidal neuron death and cognitive impairment [9, 10]. Stress impairs the structure and function of a series of brain regions [11]. The prefrontal cortex (PFC) is the main neuropathological target of stress, which is connected with numerous cortical and subcortical regions and contributes to cognitive functions [12, 13]. The dorsal medial PFC (dmPFC) includes the rostral anterior cingulate cortex and prelimbic cortex, which are the regions involved in the modulation of pain, emotions, and cognition. It has been shown that the functional inactivation of the dmPFC induces negative emotions and reduces cognitive ability [14]. The activity of dmPFC neurons conveys information about past choices and outcomes, and dmPFC removal or inactivation impairs cue guidance [15]. In addition, stress leads to the impairment of neural networks, cognitive dysfunction, hippocampal degeneration, and reduced neurogenesis [10]. The pyramidal neurons in the dmPFC and hippocampus show atrophy under the influence of stress, and psychosocial and restraint stress produce atrophy within approximately 3–4 weeks [11, 16]. The morphological changes vary according to brain region. Chronic restraint stress leads to dendritic retraction and decreases spinal density in the prelimbic area of the dmPFC and hippocampus [17]. The stress-induced reduction in hippocampal apical dendrite complexity is consistent with impaired hippocampal functions, such as learning and memory [18]. In addition, neuronal injury and apoptosis in the CA3 region lead to spatial memory deficits [19]. Behaviorally, it has been found that stress impairs various hippocampus-dependent functions, such as memory [20].

CPE is modulated under different kinds of stress and plays an important role in protecting neurons. Neurons in the hippocampus and cortex upregulate CPE expression after ischemic stress, which is related to neuronal survival [21]. In addition, after *CPE*-knockout (KO) mice received a stress paradigm when they were weaned at 3 weeks of age they exhibited CA3 region degeneration and diminished neurogenesis in the dentate gyrus (DG) [1, 22]. In this study, we used a common neuron-specific *Camk2a-Cre* system to delete neuron *CPE*. We generated a *CPE* conditional KO (cKO) mouse model to study the loss-of-function phenotype of CPE in neurons of the brain. We evaluated the effect of the stress paradigm (weaned, ear-tagged, and tail clipped) at 3 weeks of age on *CPE*-cKO mice. We used object recognition, Y maze, and fear conditioning tests at 8–10 weeks of age to evaluate cognitive performance. In addition, we analyzed the phenotypes of neurons and astrocytes, as well as neurogenesis in the hippocampus.

## Methods

### Animals

Camk2a-Cre mice and *CPE*^flox/flox^ mice were obtained from Cyagen Biosciences (Suzhou, China). All animals were housed under ambient temperature (22° ± 2 °C) with a natural light/dark cycle and allowed free access to clean water and standard rodent chow. Camk2a-Cre and *CPE*^flox/flox^ mice are referred to as *CPE*-cKO mice. At 3 weeks of age, mice were weaned, ear-tagged, and tails clipped for genotyping, causing an emotional and physical stress praradigm. We refer to the Camk2a-Cre; *CPE*^flox/flox^ mice as *CPE*-cKO, Camk2a-Cre; *CPE*^flox/−^ mice as heterozygous (HE), and *CPE*^−/−^ mice as wild type (WT). All experimental procedures for the animals were approved by the Experimental Animal Ethics Committee at the Minzu University of China.

### Polymerase chain reaction (PCR)

Tails clipped were collected to isolate DNA for PCR to identify the genotype. The primer sequences are as follows:

Flox-Forward primer: 5′-CTAAAGACACTGCATCCCTCTCTG-3′,

Flox-Reverse primer: 5′-ATGTAAGCCCACATATTGTCTCTGT-3′;

Cre-Forward primer: 5′-CATATTGGCAGAACGAAAACGC-3′,

Cre-Reverse primer: 5′-CCTGTTTCACTATCCAGGTTACGG-3′;

*CPE*-Forward primer: 5′-CTAAAGACACTGCATCCCTCTCTG-3′,

*CPE*-Reverse primer: 5′-ATGTAAGCCCACATATTGTCTCTGT-3′.

The DNA was initially denatured at 94 °C for 3 min and denatured at 94 °C for 30 s. The annealing was at 62 °C for 30 s and extended at 72 °C for 35 s. Then, the DNA was extended at 72 °C for 5 min. The products were analyzed by 1% agarose gel electrophoresis and evaluated using a Gel Image System (Tanon, Shanghai, China).

### Body weight

WT, *CPE*^flox/−^, and *CPE*^flox/flox^ mice (1–20 weeks of age; *n* = 10 for each genotype) were weighed every week, which was used to determine weight changes. The weight of each mouse was averaged for each group.

### Plasma glucose

Venous blood from the mouse tails was collected and detected by the Blood Glucose Monitoring System (Yuwell, China) after fasting for 12 h. Blood samples were drawn from the caudal vein at 0 min for fasting plasma glucose. Next, each mouse was intraperitoneally injected with a 1 g/kg glucose solution. Then, we determined the plasma glucose for 15, 30, 60, 90, and 120 min to measure blood glucose levels.

### Behavioral tests

To evaluate the behavior of WT, *CPE*^flox/−^, and *CPE*^flox/flox^ mice, 8–10-week-old animals underwent open field, object recognition, Y maze, and fear conditioning tests.

### Open field

Exploratory behavior was measured using an open-field apparatus (50 × 50 × 40 cm). Each animal was placed at one corner of the apparatus and measured for 7 min. Distance traveled was recorded for the last 5 min with a video-imaging system (Taimeng, Chengdu, China).

### Object recognition

The object recognition test was used to test learning and memory in mice. On the training day, two identical objects were placed on the diagonal of an apparatus (50 × 50 × 40 cm). The animals were allowed to freely explore for 7 min. For the testing procedure, one object was replaced with a novel object on the same diagonal as the training day. The animals were allowed to freely explore for 7 min. The time of exploration at 2–3 cm around the novel object was recorded for the last 5 min.

### Y maze

Exploratory activity and working memory were measured using a Y-maze apparatus (arm length: 30 cm, arm width: 6 cm, height of the wall: 15 cm). Each animal was placed in the central area. The number and alterations of entries into the arms were recorded for 7 min with a video-imaging system (Taimeng, Chengdu, China). The result was calculated as the correct alteration number/total arm entry number.

### Fear conditioning

The fear conditioning consisted of two chambers, and the freeze monitor box (23 × 23 × 30 cm) was placed in a larger soundproof room (30 × 30 × 37 cm). The freeze monitor box contained a metal grid for foot shock, and the vertical and horizontal movements of the animals were recorded. The first day was conditioned reflex training. The procedure was as follows: inaction for 60 s and then stimulation 12 times. The contents of stimulation included a conditioned stimulus of 30 s (75 dB), followed by a trace interval (30 s), and ended with a 2 s foot shock (30 mA) and 15 s of inaction. Six hours later, the short-term memory test was conducted, and the number of stimuli was reduced to 6. During the stimulation process, each trial consisted of the conditioned stimulus followed by a trace interval (30 s) and ended without delivery of the foot shock. The long-term memory test was carried out on the next day with an interval of one day. The method was the same as that of short-term memory to measure the quality of long-term memory.

### Nissl and immunofluorescence staining

Animals were perfused with 4% paraformaldehyde, and frozen sections of brain tissues were obtained. The brain tissues were sectioned into 30 µm sections for Nissl, doublecortin (DCX), CPE, and MAP2 staining. Rabbit anti-DCX antibody (1:1000; Cell Signaling Technology, Boston, USA, 4604S), mouse anti-CPE antibody (1:1000; BD bioscience, New Jersey, USA, 610758), rabbit anti-GFAP antibody (1:200; Cell Signaling Technology, 80788), and rabbit anti-MAP2 antibody (1:1000; Cell Signaling Technology, 8707) were used for immunofluorescence. The secondary antibodies were Alexa Fluor 594 goat anti-mouse secondary antibody (1:1000; Invitrogen, Carlsbad, CA, 11005) or Alexa Fluor 594 goat anti-rabbit secondary antibody (1:1000; Invitrogen, 11012). The relative fluorescence intensity of MAP2 and GFAP in the hippocampal Sub and dmPFC at ×100 magnification was calculated using ImageJ software (National Institutes of Health, USA).

### Western blotting

Hippocampal and PFC tissues were prepared as previously described [22]. Bands were analyzed using ImageJ software. Protein samples were run on 10% SDS-polyacrylamide gel electrophoresis gels and transferred onto nitrocellulose membranes (0.22 μm; Millipore, Billerica, MA, USA). After blocking with 5% nonfat milk, the membrane was blotted with antibodies against mouse anti-β-actin antibody (1:1000, CST, 4967S), mouse anti-CPE antibody (1:1000; BD bioscience, 610758), mouse anti-BDNF antibody (1:600, Abcam, UK, ab108319), rabbit anti-p-TrkB antibody (1:1000; Boster, Wuhan, China, BM4437), rabbit anti-AKT (1:2000, Cell Signaling Technology, 4691S), and anti-p-AKT (1:2000, Cell Signaling Technology, 23430S) overnight at 4 °C. After washing, the membranes were incubated with anti-mouse or anti-rabbit IgG antibodies for 1 h at room temperature. The protein expression level for each sample was normalized to β-actin.

### Statistical analysis

Data were analyzed by one-way analysis of variance (ANOVA) for multiple groups. Analysis was performed using GraphPad Prism 8.0 (Prism GraphPad software). All values are indicated by the means ± standard errors (SEM). *P* < 0.05 was considered significant.

## Results

### Analysis of body weight and glucose in plasma

**Fig. 1:**
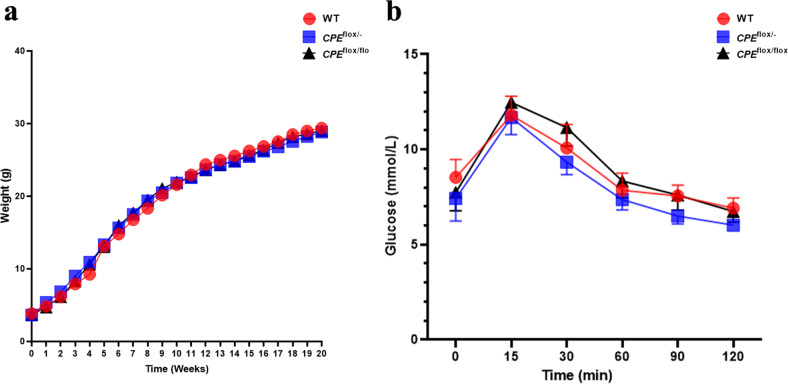
Body weight and glucose levels in *CPE*^flox/flox^ mice. **a** Weights of WT, *CPE*^flox/-^, and *CPE*^flox/flox^ mice were recorded from 1–20 weeks of age. **b** Fasting and metabolism levels of glucose in plasma of WT, *CPE*^flox/−^, and *CPE*^flox/flox^ mice. *n* = 10 from each group.

Mice aged 1–20 weeks were used for analysis of body weight and glucose in plasma (Fig. 1a). The results showed that there were no significant differences in body weight among WT, *CPE*^flox/−^, and *CPE*^flox/flox^ mice. Fasting and metabolism levels of glucose in plasma were assessed at 10 weeks of age (Fig. 1b). We found no genotype differences among WT, *CPE*^flox/−^, and *CPE*^flox/flox^ animals.

### Learning and memory loss in *CPE*^flox/flox^ mice

**Fig. 2:**
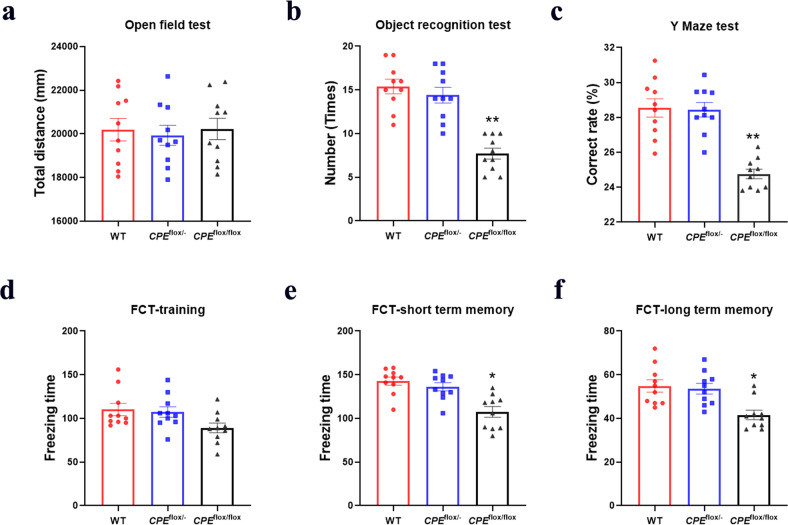
*CPE*^flox/flox^ mice show impaired learning and memory. Behavioral tests of WT, *CPE*^flox/-^, and *CPE*^flox/flox^ mice in **a** open field, **b** object recognition, **c** Y maze, and (**d**–**f**) fear conditioning tests. *n* = 10 from each group; *P < 0.05 and **P < 0.01 compared with WT; values are mean ± SEM.

From the open-field test, we observed that there was no difference among each genotype group for the distance traveled in the open-field apparatus (Fig. 2a). This indicates that the autonomous and inquiry behavior of *CPE*^flox/flox^ mice was not affected. For learning and memory in mice, we used an object recognition test (Fig. 2b). We observed that *CPE*^flox/flox^ mice spent significantly less time on novel objects than WT mice. The Y-maze and FCT-short term were carried out to assess short-term memory in mice (Fig. 2c–e). In the Y-maze test, WT and *CPE*^flox/−^ mice spent more time in the novel arm than *CPE*^flox/flox^ mice. For short-term FCT, the short-term memory retention of *CPE*^flox/flox^ mice was worse than that of WT and *CPE*^flox/−^ mice. For long-term memory retention (Fig. 2f), the fear conditioning test indicated that WT and *CPE*^flox/−^ mice had significantly better memory for foot shock than *CPE*^flox/flox^ mice.

### *CPE*^flox/flox^ mice show subiculum (Sub) degeneration

**Fig. 3:**
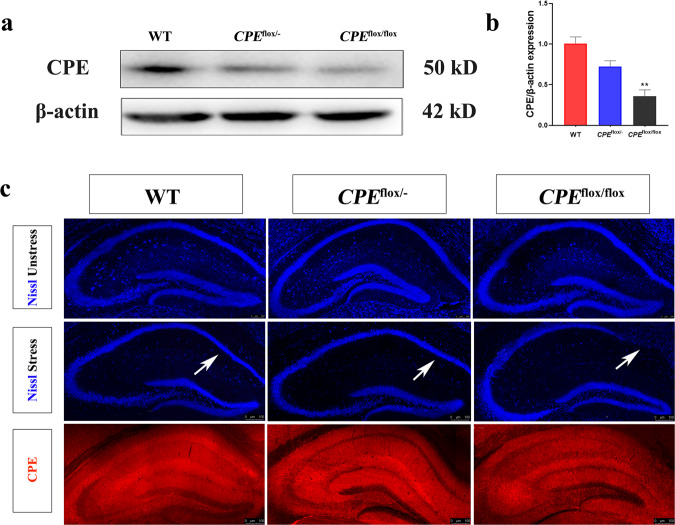
*CPE*^flox/flox^ mice show hippocampal neurodegeneration after the stress paradigm. **a**, **b** Western blot analysis of CPE levels in the hippocampus. **c** Nissl (stress and unstress) and immunofluorescence staining of CPE in the hippocampus of WT, *CPE*^flox/−^, and *CPE*^flox/flox^ mice. *n* = 6; *P < 0.05 and **P < 0.01 compared with WT; values are mean ± SEM.

We used western blotting to characterize the expression of CPE levels in the hippocampus compared with the internal control protein β-actin (Fig. 3a, b). The results showed that CPE protein in the hippocampus of *CPE*^flox/flox^ mice decreased significantly. After the stress paradigm, the Sub region of *CPE*^flox/flox^ mice was completely degenerated, but not before wearing, while the hippocampal formation of WT mice was intact (Fig. 3c).

### *CPE*^flox/flox^ mice show a reduction in GFAP and MAP2 intensity and TrkB signaling in the hippocampus

**Fig. 4:**
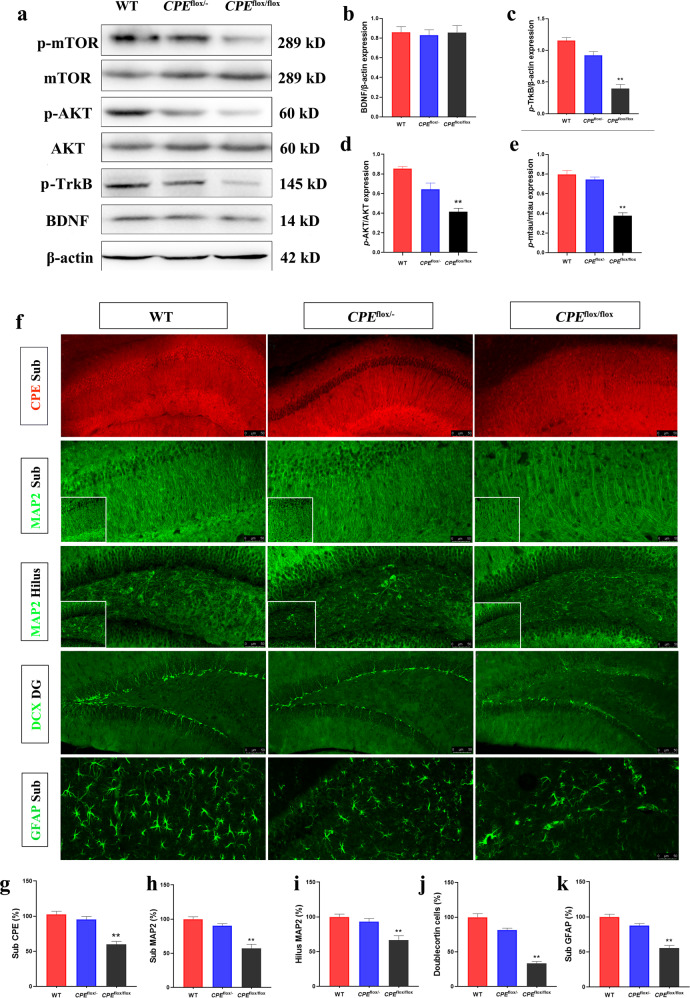
Decreased hippocampal dentate gyrus (DG) neurogenesis in *CPE*^flox/flox^ mice. **a**–**e** Western blot analysis of *p*-TrkB, BDNF, *p*-mTOR, mTOR, *p*-AKT, and AKT levels in the hippocampus. **f** Immunofluorescence of CPE, MAP2, DCX, and GFAP; and the relative fluorescent intensities of **g** CPE in Sub, **h** MAP2 in Sub, **i** MAP2 in hilius, **j** DCX in DG, **k** GFAP in DG of WT, *CPE*^flox/−^, and *CPE*^flox/flox^ mice at 100× and 400× (square in the panel). *n* = 6; *P < 0.05 and **P < 0.01 compared with WT; values are mean ± SEM.

We evaluated the expression of Akt/mTOR-mediated BDNF/TrkB pathway-related proteins. Western blot analysis of BDNF protein showed that compared with WT mice, the content of BDNF protein in the hippocampus of *CPE*^flox/flox^ mice showed equal amounts compared with WT and *CPE*^flox/−^ mice (Fig. 4a, b). *P*-TrkB protein was significantly reduced in *CPE*^flox/flox^ mice compared with WT and *CPE*^flox/−^ mice (Fig. 4a, c). The results indicated that *CPE*^flox/flox^ mice showed a decrease in hippocampal phosphorylated Akt and phosphorylated mTOR expression compared with WT mice (Fig. 4a, d, e). The immunofluorescence results showed decreased CPE expression in the hippocampal Sub (Fig. 4f, g). We used anti-MAP2 immunostaining to show MAP2 intensity in the hippocampal Sub and hilus region (Fig. 4f). The immunofluorescence results showed decreased MAP2 intensity in the Sub and hilus of *CPE*^flox/flox^ mice but not in WT and *CPE*^flox/-^ mice (Fig. 4f, h, i). In addition, DCX immunostaining showed that neurogenesis in the DG of the hippocampus in *CPE*^flox/−^ mice was similar to that in WT mice but significantly reduced in *CPE*^flox/flox^ mice (Fig. 4f, j). The GFAP intensity in the hippocampal Sub was significantly lower in the *CPE*^flox/flox^ mice than in the WT mice (Fig. 4f, k).

### *CPE*^flox/flox^ mice show a reduction in GFAP and MAP2 intensity in the dmPFC

**Fig. 5:**
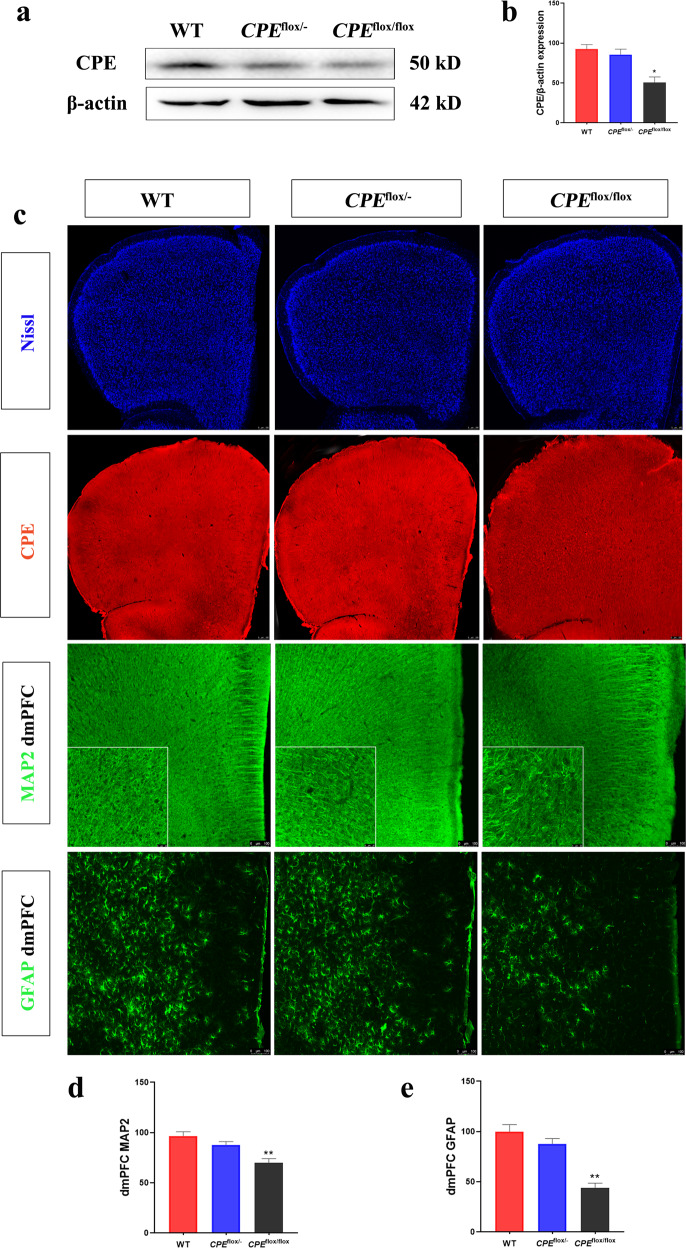
*CPE*^flox/flox^ mice show a reduction in GFAP and MAP2 intensity after the stress paradigm. **a**, **b** Western blot analysis of CPE levels in the PFC. **c** Nissl and immunofluorescence staining of CPE, MAP2, and GFAP in the dmPFC of WT, *CPE*^flox/-^, and *CPE*^flox/flox^ mice. The relative fluorescence intensities of **d** MAP2 and **e** GFAP in WT, *CPE*^flox/-^, and *CPE*^flox/flox^ mice at 100× and 400× (square in the panel). *n* = 6; *P < 0.05 and **P < 0.01 compared with WT; values are mean ± SEM.

We used western blotting to characterize the expression of CPE levels in the dmPFC compared with β-actin (Fig. 5a, b). The results showed that CPE protein in the dmPFC of *CPE*^flox/flox^ mice was significantly decreased. After the stress paradigm, the immunofluorescence results showed decreased MAP2 intensity in dmPFC *CPE*^flox/flox^ mice but not in WT mice (Fig. 5c, d). In addition, the GFAP intensity in the dmPFC was significantly lower in the *CPE*^flox/flox^ mice than in the WT mice (Fig. 5c, e).

## Discussion

In this study, we generated a *CPE*-cKO mouse to study the loss-of-function phenotype of CPE in neurons of the brain. A previous study showed that *CPE*-KO mice develop obesity and diabetes and become heavier at 8 weeks of age than WT mice [5]. Our measurements showed that there was no significant change in body weight, plasma glucose levels, and glucose tolerance of *CPE*^flox/flox^ mice compared to WT mice. Behavioral studies determined that the stress paradigm for *CPE*^flox/flox^ mice impaired learning and memory. In addition, stress and decreased CPE expression resulted in hippocampal Sub degeneration, diminished neurogenesis in the DG, and decreased neuronal density in the hippocampal Sub and dmPFC.

CPE is a prohormone-processing enzyme that processes prohormone, which cleaves the C-terminal basic residue from the precursor of enkephalin to generate enkephalin [23]. As a neuroprotective factor, CPE plays an important role in embryonic and postnatal brain development [24]. A case report indicated that a homozygous nonsense mutation in the *CPE* gene is associated with clinical phenotypes composed of obesity, intellectual disability and hypogonadism [2]. *CPE*^fat/fat^ mice show a decrease in the levels of CPE and an increase in the levels of proinsulin, which supports the role of CPE in insulin dysregulation [25]. Spontaneous point mutations in *CPE* reduce enzyme activity, leading to severe obesity [4]. *CPE*-KO mice develop diabetes, and these mice have high glucose levels at 8–10 weeks of age. The high glucose level was maintained for approximately 2 months and then began to decrease, suggesting a reversible diabetes phenotype [26]. Our study demonstrated that there was no significant difference between *CPE*^flox/flox^ and WT mice in plasma glucose levels and glucose tolerance at 10 weeks of age. *CPE*-KO mice begin to gain weight by approximately 4 weeks of age, and they are heavier than WT litters by 8 weeks [27]. It has been shown that *CPE*-KO mice show increased food consumption. The increased consumption is the result of improper signals controlling dietary behavior, involving the balance of orexigenic and anorexic neuropeptides in the hypothalamus [5]. The proper maturation of these neuropeptides requires the correct transport and processing of these neuropeptides, in which CPE plays an important role [28]. We recorded the body weight of the mice from 1 to 20 weeks, and the results showed that the deleted *CPE* in neurons did not affect body weight. In our study, *CPE*-cKO did not change weight or glucose consumption. It may be that the conditional knockout of *CPE* in neurons does not affect the signals controlling dietary behavior. Therefore, such a *CPE*-cKO mouse is a valuable model for studying the loss-of-function phenotype of CPE in neurons of the brain.

CPE plays a variety of roles in the central nervous system, including maintaining normal cognitive function, proper neuronal structure and neuronal survival [3]. CPE is thought to play a role in the cell to process precursor proteins. It also has neuroprotective activity, independent of its enzyme activity, acting extracellularly as a neurotrophic factor [25]. A novel role of CPE in neurodevelopment and the branching of proximal dendrites has been demonstrated, which is necessary for the migration and dendrogenesis of cortical neurons [29]. *CPE*-KO mice under stress lacking CPE activity exhibit many behavioral abnormalities, including learning and memory deficits [30]. Previous studies have evaluated learning and memory processes when the animals were approximately 2 months old when the weight of the mice had just begun to diverge [31].

Stress has been recognized to influence the hippocampus at various levels of analysis [32]. Behaviorally, studies have found that stress generally impairs various hippocampus-dependent learning and memory tasks [20]. Neurally, animal studies have revealed that stress alters the ensuing synaptic plasticity and firing properties of hippocampal neurons [33]. Structurally, studies have shown that stress changes neuronal morphology, suppresses the production of new granule neurons in the dentate gyrus, and reduces hippocampal volume [34]. We performed object recognition, Y maze, and fear conditioning tests on *CPE*^flox/flox^ mice at 10 weeks of age. For object recognition, WT mice showed an obvious preference for a novel object. In contrast, *CPE*^flox/flox^ mice failed to show a preference for any object during these periods. The Y maze was used to evaluate the short-term memory of mice. WT mice were more curious than *CPE*^flox/flox^ mice to explore areas that had not visited before. In addition, the fear condition test was used to evaluate short- and long-term recognition memory. WT mice were more effective in recognizing conditioned stimuli than *CPE*^flox/flox^ mice. It should be noted that conditional CPE KO mice were recently generated under the *Pomc* promotor, which knocks out cells that express proopiomelanocortin. These *CPE*-cKO mice showed normal physiology, behavior, and levels of neuropeptides [35]. In contrast, our conditional CPE KO mice under the *Camk2a* promotor showed impaired memory and neurodegeneration, supporting the hypothesis that CPE is a neuroprotective protein against stress [36].

Studies on *CPE*-KO mice have revealed multiple levels of abnormalities. Behaviorally, *CPE*-KO mice show depression-like behavior and learning disabilities. Electrophysiological analysis showed that the long-term potentiation of the hippocampus in *CPE*-KO mice was impaired. Morphological analysis showed that the hippocampus degenerated after stress when *CPE* was knocked out [31]. CPE is regulated under different kinds of stress and plays an important role in protecting neurons [3]. The effect of CPE protein deficiency on the prefrontal cortex under stress has not been reported.

There is no doubt that the behavior of learning and memory requires interaction between large-scale brain networks. Memory and recall depend on the interactions between the prefrontal cortex and hippocampus [37]. Therefore, the hippocampal-prefrontal cortex circuit plays a key role in cognitive regulation and memory consolidation [38]. In *CPE*-KO mice, the deletion of CPE protein caused injury to hippocampal CA3 neurons [39]. A previous study indicated that weaning stress upregulates glucocorticoid secretion and induces neuronal firing in DG granulosa cells to increase glutamate secretion, leading to excitotoxicity and the death of CA3 neurons in *CPE*-KO mice [40]. It may be that cKO of the *CPE* gene in neurons has a lesser effect on the endocrine system. It has also been shown that after transient global ischemia, neurons in the CA1 region were more vulnerable and only showed a transient increase in CPE expression [41]. Genetic lesions of hippocampal Sub regions have indicated distinct functions in learning and memory [42]. A classic “trisynaptic” circuit (DG > CA3 > CA1 > Sub) is a topologically ordered and partially nonoverlapping dorsal-to-dorsal, median, intermediate-to-intermediate, and ventral-to-ventral projection [43]. The main organization of mammalian hippocampal formation is a unidirectional circuit, in which the information transmitted from the surface layer of the entorhinal cortex to the DG is processed successively in the CA subfield: CA3, CA2 and CA1. Dorsal CA1 sends its main projection directly to inner entorhinal cortex layer 5 or indirectly through the dorsal Sub (a detour circuit) [42]. One of the interesting differences between direct and indirect hippocampal output pathways is that in the latter, dorsal Sub projects to entorhinal cortex layer 5 and to many cortical and subcortical brain regions [44]. Some clinical studies have shown that the DG and CA subfields are selectively activated during the formation of episodic memory, while Sub are active during the recollection of an episode [45]. In this study, the expression of CPE protein in the hippocampus and dmPFC of *CPE*^flox/flox^ mice decreased significantly. Therefore, neuronal injury and apoptosis in the Sub region lead to memory deficits. In *CPE*^flox/flox^ mice, neurological deficits may be due to partial compensatory mechanisms, including injury to hippocampal Sub and dmPFC neurons.

Astrocytes mediate developmental, physiological, and pathological processes [46]. They are considered to be the key supporting elements of neuron function, providing structural and metabolic support for neurons [47]. Astrocytes affect the recruitment and function of neurons at local and network levels [48]. We found that the number of astrocytic cells in the hippocampal Sub and dmPFC significantly decreased, possibly because CPE is important in the differentiation of neural stem cells to astrocytes [49]. BDNF is considered to be an important regulator of multiple stressors [50]. It regulates synaptic transmission and long-term potentiation in the hippocampus and participates in the formation of certain forms of memory [51]. Recently, CPE was indicated to be a critical growth and trophic factor protecting the hippocampus against stress-induced pyramidal neuron death and cognitive impairment [36]. In this study, we found that the expression of BDNF was equal to that in WT mice. This result indicates that despite the expression of similar levels of BDNF, *CPE*^flox/flox^ mice exhibit complete neurodegeneration with severe stress.

## Conclusion

In summary, this study indicates that *CPE*^flox/flox^ mice exhibit impaired learning and memory under the stress paradigm involving emotional and physical stress associated with weaning. The decreased CPE expression and stress paradigm resulted in hippocampal Sub degeneration, diminished neurogenesis in the DG and decreased neuronal density in the hippocampal Sub and dmPFC.
